# Increased self-care activities and glycemic control rate in relation to health education via Wechat among diabetes patients: A randomized clinical trial

**DOI:** 10.1097/MD.0000000000013632

**Published:** 2018-12-14

**Authors:** Yonghui Dong, Ping Wang, Zhipeng Dai, Ke Liu, Yi Jin, Ang Li, Shengji Wang, Jia Zheng

**Affiliations:** a Department of Orthopedics, Henan Provincial People's Hospital, Zhengzhou, Henan; b Department of Internal Medicine, The Second Affiliated Hospital of Hainan Medical University, Hainan Medical University, Haikou, Hainan, China.

**Keywords:** diabetes, healthy education, repeated measurement analysis, self-efficacy scale, Wechat

## Abstract

**Background:**

Health education has been considered as the effectiveness method to increase the self-care skills of diabetes patients. However, limited studies to investigate the association of health education via Wechat platform on increased the basic self-care skills and glycemic control rate in patients with type 2 diabetes.

**Methods:**

A total number of 120 type 2 diabetes patients were randomized into intervention (health education by Wechat platform plus usual care) and the control group (usual care). Biochemical parameters including fasting plasma glucose (FPG), 2-hour plasma glucose (2hPG), glycosylated hemoglobin A1c (HbA1c) were measured among the 2 groups at baseline 6-month and 12-month. Diabetes Management Self-Efficacy (SE) Scale was completed at baseline 6-month and 12-month.

**Results:**

Significant difference of HbA1c concentration and SE were found between intervention and control groups at 6-month and 12-month (*P* <.05). The effect of groups and health education duration times was found on reduced HbA1c concentration and increased the total score of SE (*P* <.05). No significant difference of FPG and 2hPG concentrations were found between intervention and control groups at 6 months and 12 months (*P* >.05).

**Conclusion:**

Health education of diabetic individuals via Wechat platform in conjunction with conventional diabetes treatment could improve glycemic control and positively influence other aspects of diabetes self-care skills.

## 1 Introduction

The prevalence of type 2 diabetes mellitus (T2DM) has been becoming a global health problem, along with the rapidly increasing percent of patients. The International Diabetes Federation estimated that there were 381.8 million people with diabetes in 2013; moreover, the number of patients would expect to increase to 591.9 million in 2035.^[1]^ In 2012, the American Diabetes Association estimated that there were more than 22.3 million people, which was equal to about 7% Americans with diagnosed diabetes in the United States.^[2]^ And the total estimated cost of people with diabetes was $245 billion in 2012.^[2]^ China has a fairly large proportion (approximately 25%) of diabetes patients in the worldwide.^[3]^ In China, the prevalence of diabetes has increased by 9.7%, which was accounted for 92.4 million adults.^[4]^ The direct economic burden of diabetes had increased in China in the past decades, for instance, the direct medical cost had reached to $9.1 billion in 2008.^[5]^

Diabetes patients were at a high risk for high morbidity and mortality rates certain disease (such as cardiovascular disease, nephropathy, neuropathy), which may due to microvascular and macrovascular dysfunction alterations.^[6]^ In China, glycemic control rate is still low, especially in elder obese patients obtained poor health education in China.^[7]^ Several studies showed that glycemic control rate of diabetes is still low, which was less than 40%.^[8,9]^ Better glycemic control could be achieved via improving the knowledge, attitude, practice, and self-efficacy (SE) of patients with diabetes. In order to prevent the occurrence of diabetes complications, people with diabetes needed to learn and maintain lifelong self-management behaviors, such as self-care activities associated to health care and daily life.^[10,11]^ It's reported that effective patient education might provide adequate information, support and monitoring that contribute to improve patients’ adherence, which in turn, decreasing the burden of diabetes chronic complications and improving the quality of life for diabetes patients.^[12–14]^

Although several studies had indicated that self-management education was related to the elevation of the glycemic control and reduce risk of diabetes complications.^[15,16]^ It is needed to develop the novel diabetes patients education programs, in order to utilize maximum limited health resources to reduce economic burden attributing by diabetes. Mobile phonebased educational interventions are considered relatively new methods, which is easily to operate and communicate with patients. Previous study had reported that a mobile health (mHealth) research platform could empower patients to take an active role in managing their own health.^[17]^ For instance, the effect of a nurse short message service intervention on glycosylated hemoglobin A1c (HbA1c) levels and adherence to treatment diabetes control recommendations was evaluated in Korean patient with diabetes patients.^[18]^

The development of mobile phone health promotion and disease prevention (including short-message service, multimedia message service, and Internet) is growing along with the widespread acceptance of cell phones. It estimated that in the worldwide, 4.55 billion people would use a mobile phone, and 1.75 billion would use a smart phone in 2014. In China, the number of mobile phone subscriber was about 1.29 billion during the period of February 2014 and February 2015. More than 90% of Chinese population used the mobile phone; due to its widely accessible technology, thus it is highly suited to imply into health interventional purposes in China. Wechat had become the most popular messaging communication app in China, which had a monthly-active-user of 549 million. Wechat platform has been implicated to solve the problems of prehospital electrocardiogram remote transmission and prehospital clinical data recording for ST-segment elevation myocardial infarction care.^[19]^ In present study, we aimed to investigate that association of health intervention based on WeChat platform, which is a new instant messaging software with altered glycemic and self-management score.

## 2 Materials and methods

### 2.1 Study design and subjects

A total number of 120 patients with T2DM aged ranged from 23 to 60 were recruited from hospitalized patients in the Second Affiliated Hospital of Hainan Medical University, during from February to May in 2016. The diabetes patients were included in this study criteria as follows: they were diagnosed T2DM aged ranged from 18 to 60 years by physicians according to World Health Organization (WHO) criteria, possessed a mobile phone, consented to receive text messages, and had the ability to read text messages and voluntarily to participate in this study. Patients were excluded if they had been diagnosed type 1 diabetes, specific diabetes, or a history of serious diseases such as renal or hepatic insufficiency, severe visual impairment, psychiatric diseases or other endocrine diseases. Each patient has been taken a physical examination, including anthropometric indices (such as height and weight) and blood biochemical test (such as blood glucose and triglyceride) according to the standard methods by physicians.

The study was approved by Medical Research Ethics Committee of The Second Affiliated Hospital of Hainan Medical University. Written informed consents were obtained from all patients before starting this study.

### 2.2 Grouping and intervention

Diabetes patients were randomly classified into control (n = 60) and intervention (n = 60) group by using a set of 120 random numbers, according to 1:1 ratio. The hospitalized diabetes patients of intervention (n = 60) group will receive conventional health education and nursing care for diabetes and will also be guided by the Wechat platform. The diabetes patients of control group will receive conventional health education and nursing care for diabetes alone. The baseline information on social and demographic characteristics (including age, gender, and educational attainment level), personal medical, and lifestyle (such as active physical activity) were collected by trained nurse of our department using the questionnaires. Self-care activities were assessed according to previously described elsewhere.^[20]^ It is a self-administered scale which is consisted of 21 diabetic items and divides into 6 self-management behaviors (general and specific diet, medication taking, exercise, blood sugar testing, foot care, and smoking). The diabetes self-care activities were asked diabetes patients in past 7 days. If they were sick in past 7 days, they were required to recall after 7 days which they were free of disease, except for diabetes. Nonsmokers scored 0, smokers scored 1; Score was calculated by individuals spend days during past 7 days on question of exercise, medication, monitoring of BG score, foot care relative items. If they spent 0, 1, 2, 3, 4, 5, 6, or 7 days on the questions of self-care activities, they scored 0, 1, 2, 3, 4, 5, 6, or 7. Additionally, other related to self-care recommendations from health care professionals’ items are also included. The Summary for diabetes self-care activities instrument was translated into Chinese and was validated by with an internal scale consistency of 0.879.^[21]^

### 2.3 Follow-up and outcome indicators

The physicians provided and selected the educational related messages and trained the nurses who sent the educational text messages and collected question of the diabetes patients. Diabetes patients in the intervention groups were classified into 5 groups and followed up by trained nurse via established Wechat platform. They had gained the college degree or above certificate and had worked at least 3 years. They were responsible for sending and explaining the diabetes related-knowledge including self-monitoring of blood glucose, reasonable diet, exercise prescription, compliance with prescribed medication, and management of low and high blood sugar, as well as weight control for patient with diabetes. The communicated frequency between nurse and patients was 3 to 5 times in the first week, 2 to 4 times in the second week, and 1 time from in the third week to the end follow up. Data information on self-care activities were collected from all patients at baseline, 6 months and 12 months. The fasting plasma glucose (FPG) and 2-hour plasma glucose (2hPG) and glycosylated hemoglobin (HbAlc) were measured at baseline, 6 months and 12 months.

### 2.4 Statistical analysis

For the analyses, the mean (standard deviation) and proportions were used to express the normal distribution of variables and categorical variables respectively. Chi-square test was used to analyze differences in the categorical variables. Repeated-measures analyses of variance were conducted to assess the alter blood glucose and the self-care activities score between innervation groups and control groups. All data were double-keyed and analyzed using EpiData 3.0 software (EpiData Association Odense, Denmark) and SPSS 12.0 software (SPSS Inc. Chicago, IL), respectively. A 2-tailed *P* value no more than .05 was considered as statistical significance.

## 3 Results

### 3.1 Baseline characteristics

A total number of 120 diabetic individuals were included in this study (60 patients for each group) with a mean age of 42.7 ± 6.7 (range 18–60years). One diabetes patient was not followed up, due to sudden death. The proportions of male and female were 52.1% and 47.9 respectively. The duration of diabetes years was ranged from 1.5 to 17 years. The frequency of SMBG (≥ 7 times) in intervention group patients was higher than control group patients. There were no significant differences of age and educational levels between control group and intervention group. There were no significant differences of sex, family history of diabetes, body mass index (BMI), diabetes mediation, and complications between the intervention and control groups (Table 1).

**Table 1:**
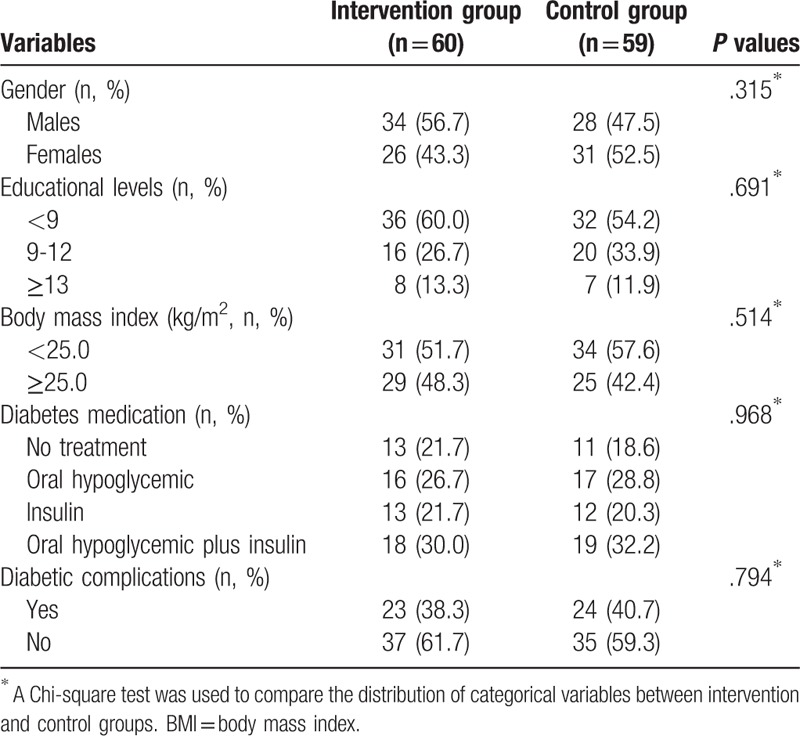
Distributions of selected variables of patients between intervention and control group include sex, family history of diabetes, BMI, diabetes mediation, and complications.

### 3.2 Wechat altered participants blood glucose level

As shown in Table 2, no significant difference of FPG and 2hPG between the intervention and control group at baseline, 6-month and 12-month; but the levels of FPG and 2hPG was accompanied with time declined among intervention group (*P* <.01) and 2hPG was also accompanied with time reduced in the control group (*P* <.05). While significant difference of HbA1c between intervention and control groups was found at 6-month and 12-month (*P* <.05). The HbA1c concentration was declined along with followed up time among both groups and the intervention group had lower HbA1c concentration than the control group. The interaction between groups and times was found on the HbA1c concentration (*P* <.05). There was no significant difference BMI between at baseline, 6 months and 12 months.

**Table 2:**
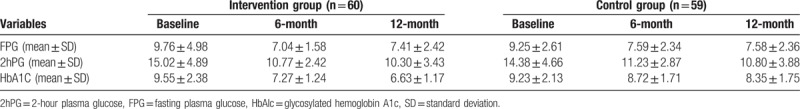
Altered fasting plasma glucose, 2-hour plasma glucose, and glycosylated hemoglobin A1c between intervention and control groups.

### 3.3 Difference of the SE score

As shown in the Table 3, the total score of SE and the score of foot care, medication taking, and diet were higher in the intervention group than the control group at 6-month and 12-month. No significant difference the score of exercise, monitor of blood glucose and smoking between the intervention and control groups. However, the total score of SE and the score of foot care, medication taking, diet, exercise, monitor of blood glucose were increased as the follow-up increase (*P* <.05). The interaction between groups and times was found among the variables of total score of SE and the score of foot care and diet (*P* <.05).

**Table 3:**
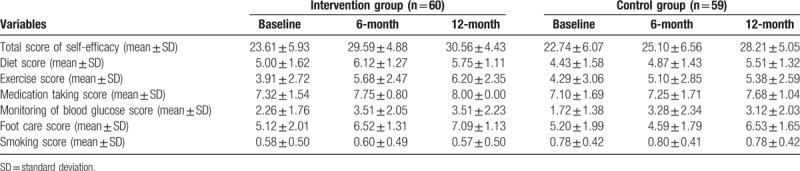
Difference of the self-efficacy score between intervention and control groups in the study participants, include total score of self-efficacy, diet score, exercise score, medication taking score, monitoring of blood glucose score, foot care score, and smoking score.

## 4 Discussion

In present study, we found that diabetes patients had a higher HbAlc concentration decreased and higher total score of SE scale in intervention group than the control groups, implicated that Wechat was an effective way to increase glycemic control rate and elevate diabetes patients through Wechat platform sending diabetes-related educational message.

Although, the FPG measurement has been widely available and inexpensive used to diagnose diabetes,^[22]^ a growing evidence had suggested that HbA1c had been considered as a diagnostic criterion for diabetes and measured the HbA1c concentration including several advantages such as need fasting samples, less biological variability and reflecting over a 3-month period of average blood glucose.^[22,23]^ Moreover, study showed that the variability within-person of HbA1c (within-person coefficient of variation (CV): 3.6%; 95% CI: 3.2, 4.0) was lower than 2hPG (CV: 16.7%; 95% CI: 15.0, 18.3) or FPG (CV: 5.7%; 95% CI: 5.3, 6.1).^[24]^ In addition to biological variability, plasma glucose levels were affected by glycolysis, which may reduce its levels before analysis. Although the preservative sodium fluoride had no effect on the reduction in plasma glucose in the first hour after sample collection, it appeared to slow glycolysis considerably in the second hour.^[25]^ Thus, the results indicated that HbA1c may be more reproducible than 2hPG or FGP. Taken those together, HbA1c is more sensitive to reflect the long-term of blood glucose variation.

We observed that HbA1c levels were lower intervention groups via Wechat platform health education than that control groups. Several studies showed that mobile phone based health education had been suggested that nonsignificant differences in HbA1c between intervention and control groups.^[26,27]^ Those studies of mobile phone based education had used passive collected the monitoring of blood glucose.^[26,27]^ The reason might be that, in this study, diabetic individuals were educated via multiple methods (such as voice and video chat and send text messages) by using Wechat platform. It was convenient, costless and easy for to communicate with educators and ask questions about self-management of diabetes. Moreover, diabetic patients were followed up by telephone in several times to confirm diabetic individuals reading the related educational messages and recruit them to the hospital for health examination. In contrast, some studies had been reported that mobile phone-based health education had statistically significant improvements in HbA1c levels between intervention and control groups.^[28,29]^ Evidence had been demonstrated that internet it was effective at improving glycemic control rate and diabetes knowledge compared to usual care among diabetes patients.^[30]^

Our result showed that sending diabetes-related health education messages via Wechat platform to diabetes patient had lower HbA1c levels than those without receiving health education messages. A cross-sectional study showed that self-care behaviors (such as appropriate diet, regular exercise, and medication) might be contributed to achieve effective diabetes control and maintain it in type 2 diabetes patients in Taiwan.^[31]^ Evidence showed that HbA1c level and receiving diabetes education were related to self-care activities except for smoking among individuals with type 2 diabetes in Turkey.^[32]^ Our results showed that the total score of SE scale was negatively associated with HbA1c level, which indicated that diabetic individuals received the diabetes-related health education messages had increased the awareness to monitor their blood glucose levels. Previous study had shown that sending short text messages in conjunction with conventional diabetes treatment could improve glycemic control which might through influence other aspects self-care of diabetes patients.^[15]^

Wechat platform was used to health education including several advantages: first it could not only make patients to receive the treatment suggestion from the physician, and also reduce the round-trip time, which indirectly reduces the economic burden and convenience to diabetic individual; second, compared to mobile phone short message service, it was cheaper, widely and easily to accept by diabetes; third, it could increase the interaction of doctor-patient and not only benefit to individual and also affect individuals outside the group via the circle of friends can also affect individuals outside the group. Moreover, it could increase chances of diabetes patients to gain the professional knowledge of diabetes and communicate with the physician who contributed to strengthen the self-care consciousness and promote to change the unhealthy habits.

Several strengths needed to be mentioned: first, this study is first used Wechat platform to educate the diabetic individuals by improving their self-care skills; second, Wechat was a costless, simple and accessible method for diabetic individuals to accept; third, diabetic individuals could share diabetes health education related message in their friends circle, which might had benefit by affecting their friends life habits to manage their blood glucose. There are several limitations needed to be addressed. First, data information on self-care activities were obtained by self-reporting and may induce biases by recall; second, the diabetic subjects were invited from one single hospital inpatients and could not be generalized those results to all Chinese people with type 2diabetes.

In conclusions, health education diabetes patients via WeChat platform might be effective to elevate their self-care activities awareness and reduced their HbA1c level, which might be contributed to decrease the diabetes-related complications.

## Acknowledgment

The authors are grateful to all volunteers who participated in this study.

## Author contributions

**Conceptualization:** Yonghui Dong, Ping Wang, Jia Zheng.

**Data curation:** Yonghui Dong, Zhipeng Dai, Ke Liu.

**Formal analysis:** Yonghui Dong, Yi Jin, Ang Li, Jia Zheng.

**Funding acquisition:** Yonghui Dong, Ping Wang, Jia Zheng.

**Investigation:** Yonghui Dong, Ping Wang, Shengjie Wang.

**Methodology:** Yonghui Dong, Ping Wang, Zhipeng Dai, Jia Zheng.

**Project administration:** Yonghui Dong, Zhipeng Dai, Ke Liu, Jia Zheng.

**Resources:** Ke Liu.

**Software:** Ang Li, Shengjie Wang.

**Supervision:** Yonghui Dong.

**Validation:** Yonghui Dong.

**Visualization:** Yonghui Dong, Ping Wang.

**Writing – original draft:** Yonghui Dong, Ping Wang.

**Writing – review & editing:** Yonghui Dong, Ping Wang, Jia Zheng.
